# Interfacial Chemistry of Phosphate-Functionalized Self-Doped TiO_2_ Nanotube for Electrochemical Detection of Methylene Blue

**DOI:** 10.1021/acsomega.6c02245

**Published:** 2026-06-02

**Authors:** Victor Lorejan Pinto, Rafael A. L. Chioquetti, Silvia H. P. Serrano

**Affiliations:** Department of Fundamental Chemistry, Institute of Chemistry, 153989 University of São Paulo, Av. Prof. Lineu Prestes, 748, 05508-000 São Paulo, Brazil

## Abstract

Titanium dioxide nanotube (TNT) arrays were synthesized by electrochemical anodization of Ti sheets and subsequently modified by cathodic polarization at −1.5 V in phosphate buffer (pH 7.4), yielding self-doped TiO_2_ nanotubes (SD-TNT). This treatment generated Ti­(III) states, enhancing conductivity and surface hydrophilicity while also promoting phosphate ion adsorption. Structural and morphological analyses confirmed the ordered nanotubular architecture, while electrochemical studies revealed improved charge-transfer properties. In acidic medium (pH 2.0), SD-TNT electrodes exhibited pronounced interaction with protonated methylene blue (MB^2+^), favoring its accumulation on the electrode surface. Cyclic voltammetry revealed distinct kinetic regimes depending on the scan rate, with Tafel analysis at low scan rates identifiying the second electron transfer as the rate-determining step, while resistive and capacitive effects became dominant at higher scan rates. The synergistic effect of self-doping and phosphate functionalization modulated the interfacial behavior of SD-TNT, enabling sensitive and reproducible electrochemical detection of MB^2+^. These findings highlight the role of surface chemistry in tuning charge-transfer processes and demonstrate the potential of SD-TNT as a model platform for interfacial electrochemistry studies and environmental sensing applications.

## Graphical abstract

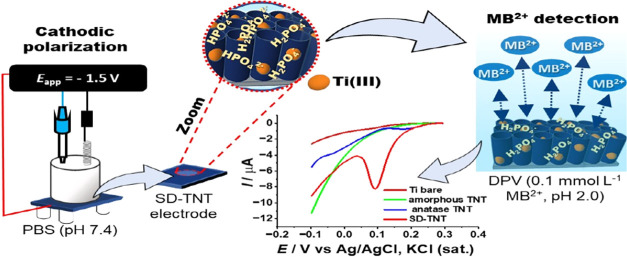

## 1 Introduction

Water security and the sustainable management of aquatic resources have become central issues in environmental research. In this context, emerging pollutants represent a growing concern, as they have been increasingly detected in surface water, groundwater, food, and other resources.^1,2^ These contaminants mainly originate from insufficient wastewater treatment associated with agricultural, industrial, and domestic activities.^3,4^ Because EPs are not yet comprehensively regulated or systematically monitored, they pose a significant challenge to environmental management.^5^ This group includes pharmaceuticals, personal care products, endocrine-disrupting chemicals, hormones, pesticides, and synthetic dyes.^3,6^

Among these pollutants, synthetic dyes deserve particular attention. They are widely employed in industrial applications such as textiles, cosmetics, leather, food, paints, varnishes, and pulp and paper production.^7–9^ Methylene blue (MB) is one of the most frequently detected dyes in industrial effluents, particularly from textile and dyeing facilities.^10^ MB is recalcitrant and resistant to natural degradation. Even at trace concentrations, it alters the visual appearance of water, hinders light penetration, and compromises the photosynthetic process. Consequently, the metabolic activity of aquatic organisms is affected, leading to oxygen depletion in water resources.^11^ Moreover, conventional wastewater treatments are often ineffective for dye removal, creating risks to ecosystems.^12,13^ Beyond its environmental persistence, MB poses direct toxicological risks to both humans and animals upon sustained contact.^10^

Electrochemical techniques have emerged as powerful tools for monitoring and understanding the behavior of redox-active pollutants in aqueous environments. Compared with conventional analytical methods, electrochemical approaches offer high sensitivity, operational simplicity, and real-time detection capabilities while providing valuable insights into interfacial charge-transfer processes.^14,15^ In this context, the design of robust and stable electrode materials is critical for achieving both analytical reliability and fundamental understanding of pollutant and surface interactions.

Titanium emerges as a viable alternative: it combines chemical stability, corrosion resistance, and affordability.^16^ Importantly, the anodization of titanium yields highly ordered TiO_2_ nanotube (TNT) arrays, characterized by a large surface area and direct electron pathways, which eliminate the need for conductive binders.^17^ Nevertheless, pristine TNTs exhibit limited electrochemical activity due to their semiconducting nature and wide band gap (∼3.2 eV), which restricts electron-transfer kinetics and reduces sensitivity in electroanalytical applications.^18^

Further modification of TNTs has enabled the development of new functionalities. A particularly relevant strategy is the preparation of self-doped TiO_2_ nanotubes (SD-TNT) by cathodic polarization, which reduces Ti­(IV) to Ti­(III) through proton intercalation.^12,19–23^ The formation of Ti­(III) states narrows the band gap, enhances conductivity, and imparts metallic-like electrochemical behavior, all without the use of external dopants.^24,25^ This approach is simple, cost-effective, and highly versatile compared with traditional doping strategies. Although the Ti­(III) states are inherently metastable and susceptible to reoxidation upon exposure to ambient air or oxidizing aqueous environments,^26^ a thorough understanding of the electrochemical behavior of SD-TNT is essential to establish reproducible fabrication conditions, including controlled overpotentials and standardized polarization parameters, that ensure consistent electrode performance. Such knowledge provides a reliable foundation upon which surface functionalization strategies can be systematically explored.

In addition to electronic modification, surface functionalization also plays a crucial role in controlling the interfacial behavior of TiO_2_-based electrodes. Phosphate functionalization of TiO_2_ has been widely explored in photocatalytic degradation and water oxidation processes, for instance.^27,28^ This is because phosphate ions are known to strongly adsorb onto TiO_2_, leading to significant modifications in its interfacial chemistry, such as the formation of a negative electrostatic field induced by phosphate anions.^29–32^ In this context, investigating the electrochemical behavior of cationic and environmentally relevant species, such as MB, at the TiO_2_ electrode interface becomes particularly appealing, as this phenomenon remains poorly understood.

In this work, TiO_2_ films obtained by anodization on Ti substrates were subjected to cathodic polarization in a phosphate buffer solution (PBS). The present study has two main objectives: (i) to investigate the morphological, structural, and electrochemical modifications induced by the self-doping process and by phosphate interaction at the TiO_2_ interface and (ii) to elucidate the interaction mechanisms between MB and the SD-TNT surface. The insights obtained underscore the potential of SD-TNT as a platform for the electrochemical sensing of MB in aqueous environments.

## 2 Experimental Section

### 2.1 Reagents and Solutions

All chemicals used were of analytical grade and employed without further purification. K_3_[Fe­(CN)_6_] was purchased from Merck, [Ru­(NH_3_)_6_]­Cl_3_ from Sigma-Aldrich, and MB from Synth. Stock phosphate buffer solution (PBS), pH 7.4, was prepared by dissolving Na_2_HPO_4_ (Merck) and NaH_2_PO_4_ (Merck) in deionized water to obtain a total phosphate concentration of 1.0 mol L^–1^. PBS at 0.1 mol L^–1^ and pH 2.0 was prepared by adjusting an H_3_PO_4_ solution with 2 mol L^–1^ NaOH. Britton–Robinson (BR) buffer of 0.04 mol L^–1^ was prepared by dissolving the appropriate amount of sodium borate and diluting the appropriate volume of phosphoric and acetic acids in deionized water. pH measurements were carried out at 25 °C using a pH meter equipped with a combined glass electrode (Metrohm, model 654).

### 2.2 Preparation of TNT and SD-TNT Electrodes

Titanium sheets (99.2% purity, 1.6 mm thickness, Hypertriton – Canada) were previously cut (1.0 cm × 2.0 cm), mechanically polished with different sandpapers, and degreased by ultrasonication in isopropyl alcohol, acetone, and ultrapure water for 15 min each. They were then chemically treated using a solution containing HNO_3_ (5% v/v) and HF (10% v/v) for 1 min, rinsed with deionized water, and dried in a nitrogen flow. The TNT electrodes were prepared by anodization (potentiostat Hikari HF-3203S) using a two-electrode cell configuration, with Ti as the anode and Ag (1 cm^2^) as the cathode. The electrolyte consisted of 0.25% NaF and 10% water in glycerol at a constant potential of 20 V (sweep rate of 2.0 V min^–1^) for 48 h. The distance between the electrodes was fixed at 3.0 cm. Finally, TNT was calcined at 450 °C using a tubular furnace EDG FT-HI/20 (sweep rate of 20 °C min^–1^) for 1 h to transform amorphous TiO_2_ into crystalline anatase phase.

SD-TNT electrode (geometric area: 0.071 cm^2^) was prepared by cathodic polarization at a constant potential of −1.5 V for 15 min in 1 mol L^–1^ PBS, pH 7.4. Ag/AgCl, KCl (sat.) as a reference electrode, and platinum (Pt) as a counter electrode were used in a conventional three-electrode system (Autolab PGSTAT 10 potentiostat with NOVA 1.11 software).

### 2.3 Analytical Curves

Differential pulse voltammograms (DPVs) were recorded in duplicate with the following parameters: modulation amplitude of 0.025 V, step potential of −0.005 V, and scan rate of 0.010 V s^–1^. Analytical curves were prepared using the SD-TNT electrode preconditioned at 0.3 V for 10 min in 0.1 mol L^–1^ PBS (pH 2.0), containing 0.5 mol L^–1^ Na_2_SO_4_. Prior to each measurement, the solutions were deaerated by purging with nitrogen gas. MB concentrations in the electrochemical cell ranged from 1 × 10^–5^ to 9 × 10^–5^ mol L^–1^ in the same electrolyte and pH. For the calculation of the detection (LOD) and quantification (LOQ) limits, eqs 1 and 2 were considered.^33^

1 $$\mathrm{LOD}=\frac{{3s}_{B}}{m}$$

2 $$\mathrm{LOQ}=\frac{{10s}_{B}}{m}$$

where *s*_B_ is the standard deviation of the blank current (taken from 10 DPV measures of blank) at the same *E*_p_ values as measured in the presence of the analyte and m is the slope of the analytical curve.

### 2.4 Material Characterization

The morphology of TNT was studied using a scanning electron microscope (SEM) JSM-7401F (JEOL) equipped with a field-emission electron gun. Energy-dispersive X-ray spectroscopy (EDS) analysis was performed at 30 kV using an EDS detector attached to the SEM. X-ray diffraction analysis (Bruker D2Phaser) was performed for chemical characterization. Contact angle measurements were performed by depositing 10 μL of water on titanium-based electrodes inside a monitored chamber maintained at 25 ± 0.5 °C.

The electrochemical measurements were performed with a potentiostat/galvanostat PGSTAT 10 (Metrohm Autolab), controlled with NOVA 1.11 (Metrohm Autolab). The first cycle of each voltammogram was used for data analysis. Double-layer capacitance (*C*_dl_) values were determined by recording cyclic voltammograms (CVs) at different scan rates (ν) from 0.020 to 0.140 V s^–1^. The potential used to obtain the *C*_dl_ in CVs at different ν was 0.05 V. The *C*_dl_ was calculated from *I*–ν plot according to eq 3

3 $$I=\nu C_{\mathrm{dl}}$$

where the slopes of the *I*–ν plot correspond to the *C*_dl_ values.

Furthermore, to characterize the electrochemical behavior of MB on the SD-TNT electrode, Tafel analysis was performed.^34^

## 3 Results and Discussion

### 3.1 Characterization of TNT and SD-TNT Electrodes

Figure 1 shows the morphologies of TNT electrodes produced via anodization of Ti sheets after 2 h (Figure 1a) and 48 h (Figure 1b). Initially, a compact oxide layer is formed under the applied potential, eq 4. Uniformly distributed pores are formed on the surface. Finally, chemical dissolution of titanium oxide occurs according to eq 5.^35,36^

4 $$\mathrm{Ti}\left(s\right)+2H_{2}O\left(l\right)\to {\mathrm{TiO}}_{2}\left(s\right)+4H^{+}\left(\mathrm{aq}\right)+4e^{-}$$

5 $${\mathrm{TiO}}_{2}\left(s\right)+6F^{-}\left(\mathrm{aq}\right)+4H^{+}\left(\mathrm{aq}\right)⇌{{\left[{\mathrm{TiF}}_{6}\right]}^{2}}^{-}\left(\mathrm{aq}\right)+2H_{2}O\left(l\right)$$

**Figure 1:**
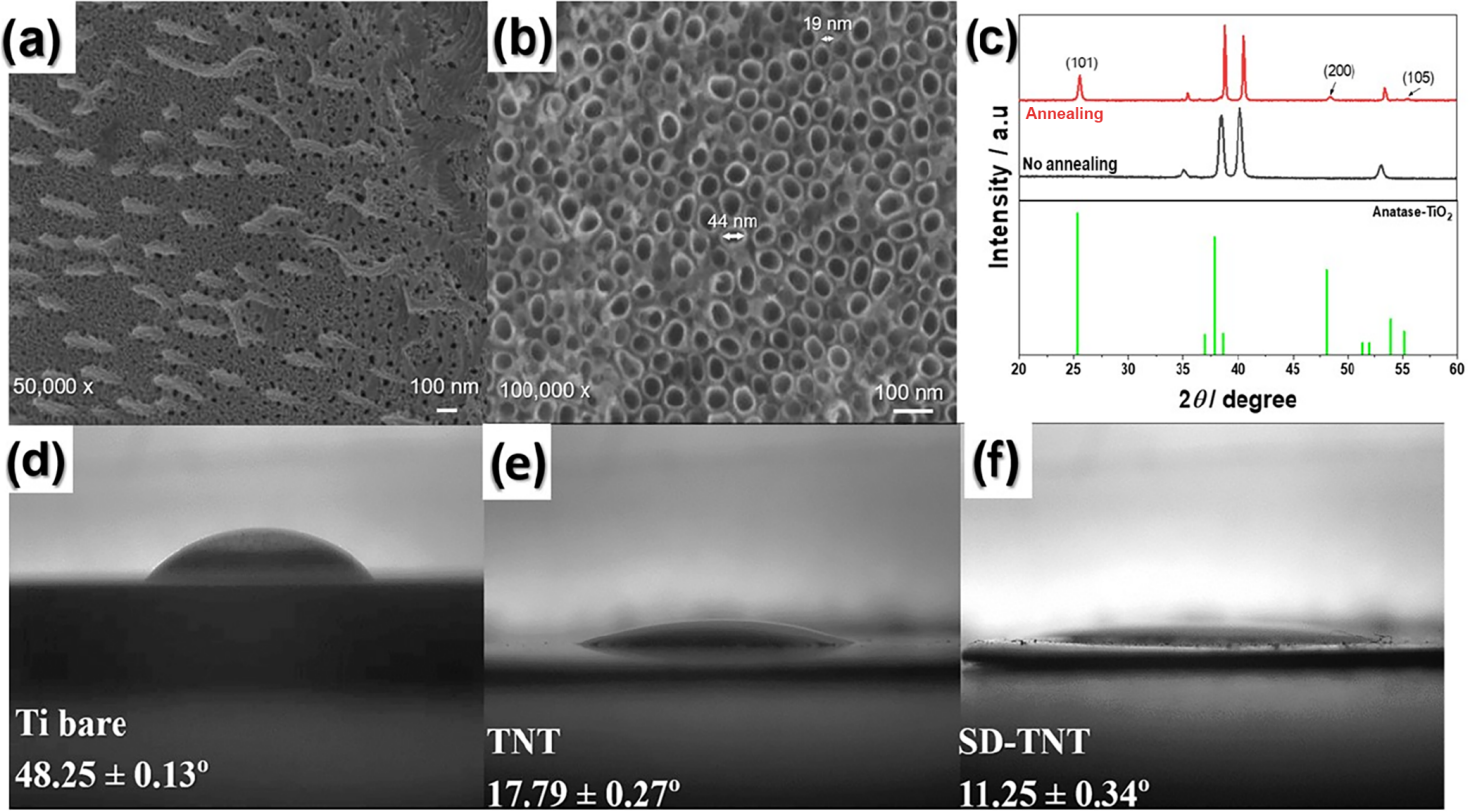
SEM images of the TNT surface after 2 h (a) and 48 h (b) of anodization. XRD patterns of annealing (red line) and nonannealing TNT (black line). The green bars are indexed to the anatase phase (JCPDS 71-1167) (c). Sessile drop assay with deionized water drop on bare Ti (48.25°) (d), TNT (17.79°) (e), and SD-TNT (11.25°) (f).

Figure 1b suggests that after 48 h the anodization process promoted the growth of a highly ordered nanotube structure with mean inner diameters varying from 19 to 44 nm. EDS elemental analysis showed that the atomic ratio between Ti (31.10%) and O (62.34%) was approximately 1:2, confirming the formation of anodized TiO_2_.

Figure 1c shows the XRD patterns of unannealed and annealed TNT. The amorphous TNT on Ti substrate shows only peaks corresponding to Ti. A crystallized TNT structure was observed after annealing, with peaks 25.3, 48.4, and 55.1° identified on (101), (200), and (105) anatase crystal faces, respectively (Joint Committee on Powder Diffraction Standards, JCPDS 71-1167).

The wetting of bare Ti, TNT, and SD-TNT electrodes can be observed in Figure 1d. The bare titanium had the largest contact angles with 48.25 ± 0.13°. The formation of the TNT structure made the surface more hydrophilic (17.79 ± 0.27° contact angle), with a contact angle decrease of 37% compared to the bare Ti. TiO_2_ is expected to be more hydrophilic than bare Ti due to the ability of water molecules to form hydrogen bonds with hydroxyl groups present on the oxide surface. Additionally, the surface morphology contributes to an even lower contact angle due to the enlarged gap between nanotubes, which allows greater water penetration. Furthermore, the annealing treatment produces anatase-phase nanotubes, which contribute to increase the hydrophilicity.^37,38^ Finally, the SD-TNT electrode exhibited a lower contact angle (11.25 ± 0.34°), indicating greater hydrophilicity.

### 3.2 Electrochemical Characterization of the TNT and SD-TNT Electrodes

Figure 2a demonstrates the *C*_dl_ values obtained from the current vs ν plots for TNT and SD-TNT electrodes during CVs at ν ranging from 0.020 to 0.140 V s^–1^ at 0.05 V vs Ag/AgCl (KCl, sat.). Figure S1 shows the differences between TNT and SD-TNT in CV obtained after applying −1.5 V for 15 min. First, the CVs of the TNT and SD-TNT electrodes clearly exhibit a large integrated area (inset, Figure 2a). This electrochemical behavior is indirectly associated with a high effective surface area,^39^ as suggested by the high density of nanotubes observed in the SEM images (Figure 1b).

**Figure 2:**
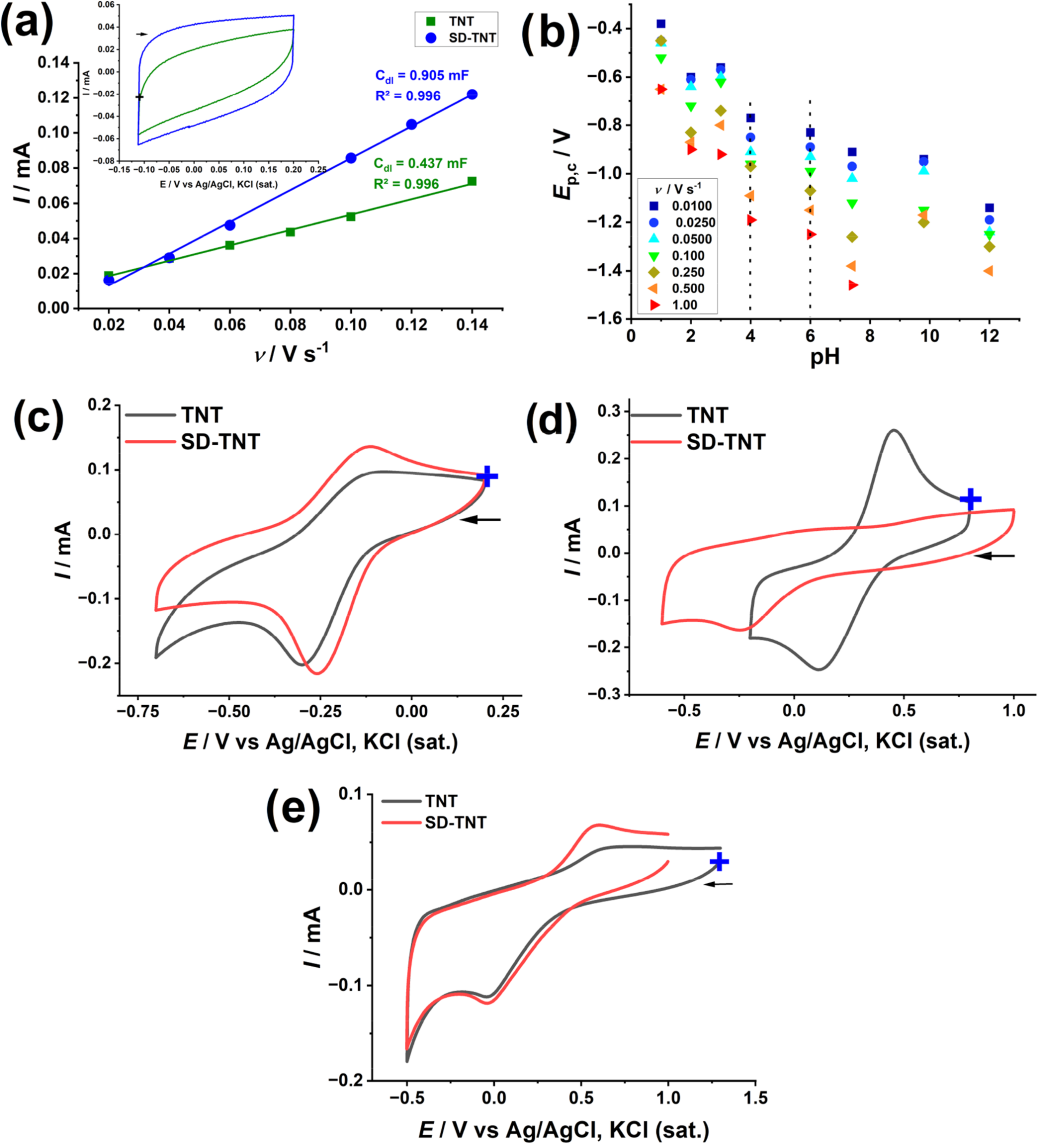
Capacitive current (*I*) measured at 0.05 V vs Ag/AgCl (KCl sat.) for TNT and SD-TNT as a function of scan rate ν. *C*_dl_ values were obtained from linear fits. Inset: CVs of TNT (green line) and SD-TNT (blue line) recorded from −0.10 to 0.20 V vs Ag/AgCl (KCl sat.) at 0.100 V s^–1^ in 0.5 mol L^–1^ H_2_SO_4_ (a). *E*_p_ vs pH plot of TNT in 0.1 mol L^–1^ PBS (pH 2.0–12) from 0.010 to 1.0 V s^–1^ (b). CVs in KCl 0.5 mol L^–1^ containing 5 mmol L^–1^ ⌈Ru­(NH_3_)_6_⌉^3+^ (c) and 5 mmol L^–1^ [Fe­(CN)_6_]^3–^ (d) at 0.100 V s^–1^. CVs of TNT (before) and SD-TNT (after cathodic polarization in 1 mol L^–1^ NaClO_4_), recorded in 0.5 mol L^–1^ KCl containing 5 mmol L^–1^ [Fe­(CN)_6_]^3–^ at 0.100 V s^–1^ (e).

From a geometrical perspective, highly ordered nanotube arrays on a metallic substrate can provide direct pathways for electron transfer.^40^ The growth of TiO_2_ nanotubes on metallic titanium (TNT electrodes) enhances the conductivity of titania. Additionally, the surface morphology of TNT promotes a high capacitance by facilitating the transfer of solvated ionic species into the nanopores.^40,41^ Moreover, the reduction of Ti­(IV) to Ti­(III) via cathodic polarization resulted in additional modifications in the electrochemical behavior of the SD-TNT electrode.

The capacitance of SD-TNT (0.905 mF) is significantly higher than that of TNT (0.437 mF) (Figure 2a). A similar increase in capacitance has also been reported in the literature.^19,20,42^

After cathodic polarization, the SD-TNT electrode exhibited lower resistance as compared to TNT, which can be seen by comparison with simulated data based on the uncompensated resistance (*R*_u_) (Figure S2).

Under cathodic polarization, electron injection to nanostructured TiO_2_ is accompanied by charge-compensating proton intercalation (eq 6).^43^ The intercalation of H^+^ and formation of donor species Ti­(III) create oxygen vacancies (defects) in the titania structure. An increase in these defects generates free electrons, which reduce electrical resistance.^44,45^ Complete modification is achieved upon uniform Ti­(III) formation and H^+^ incorporation throughout the nanotubes, with reduction initiating at the tube bottoms and progressively extending to the walls, which ultimately dominate charge transport due to their higher conductivity.^46^

6 $${\mathrm{Ti}}^{\mathrm{IV}}O_{2}+e^{-}+H^{+}⇌{\mathrm{Ti}}^{\mathrm{III}}\left(O\right){\left(\mathrm{OH}\right)}^{-}$$

The reduction of Ti­(IV) to Ti­(III) is strongly pH-dependent, as it involves coupled electron transfer and proton intercalation (eq 6). As shown in Figure 2b, the cathodic peak potential (*E*_p,c_) shifts to more negative values with increasing pH and scan rate. The pH dependence reflects the role of proton availability, with lower pH favoring proton intercalation and shifting the reduction to more positive potentials. Cyclic voltammograms were recorded over a pH range from 1.0 to 12 in 0.1 mol L^–1^ electrolyte (phosphate buffer where applicable; Figure S3).

At a given pH value, *E*_p,c_ shifts with an increasing scan rate (Figure 2b), indicating that the cathodic process described by eq 6 is not electrochemically reversible. In other words, at higher scan rates, the experimental time scale becomes too short to sustain proton intercalation and Ti­(IV) reduction, leading to increasing deviation from equilibrium and progressively more negative peak potentials. Indeed, the literature reports that proton intercalation is relatively slow.^46^

Figure 2c,d shows the CVs obtained with TNT and SD-TNT electrodes in the [Fe­(CN)_6_]^3–^ and [Ru­(NH_3_)_6_]^3+^ probe solutions. Equations 7 and 8 represent the reduction process of the probes, each involving one electron.

7 $${\left[\mathrm{Fe}{\left(\mathrm{CN}\right)}_{6}\right]}^{3-}+e^{-}⇌{\left[\mathrm{Fe}{\left(\mathrm{CN}\right)}_{6}\right]}^{4-}$$

8 $${\left[{\mathrm{Ru}\left({\mathrm{NH}}_{3}\right)}_{6}\right]}^{3+}+e^{-}⇌{\left[{\mathrm{Ru}\left({\mathrm{NH}}_{3}\right)}_{6}\right]}^{2+}$$

Initially, it can be observed that cathodic polarization in a 1 mol L^–1^ PBS solution at pH 7.4 resulted in the highest electrochemical performance in the [Ru­(NH_3_)_6_]^3+^ solutions (Figure 2c), in contrast to the behavior observed for [Fe­(CN)_6_]^3–^ (Figure 2d). The reversibility criteria values (Table 1) confirm that the peak-to-peak separation (*E*_p,a_ – *E*_p,c_) decreased from 178 to 125 mV for [Ru­(NH_3_)_6_]^3+^, while the ratio of cathodic to anodic current (*I*_p,a_/*I*_p,c_) increased from 0.35 to 0.73.^34,47,48^ Conversely, the anodic process was poorly defined in the reverse scan obtained in the solution containing [Fe­(CN)_6_]^3–^, indicating that the SD-TNT surface acquires a net negative charge, which hinders the approach of negatively charged species due to electrostatic repulsion.

**Table 1:** Reversibility Criteria Obtained from Cyclic Voltammograms Using TNT and SD-TNT in the Presence of [Fe­(CN)_6_]^3–^ and ⌈Ru­(NH_3_)_6_⌉^3+^

electrode/probe	*E*_p,a_ – *E*_p,c_ (in mV)	|*I*_p,a_/*I*_p,c_|
TNT/[Fe(CN)_6_]^3–^	340	0.89
SD-TNT/[Fe(CN)_6_]^3–^		
TNT/⌈Ru(NH_3_)_6_⌉^3+^	178	0.35
SD-TNT/⌈Ru(NH_3_)_6_⌉^3+^	125	0.73

According to well-established studies, phosphate species readily adsorb onto titania.^30,49,50^ Therefore, it is highly likely that phosphate species (H_2_PO_4_^–^ and HPO_4_^2–^) were adsorbed onto the SD-TNT surface, resulting in an electrode with a surface net negative charge. Consequently, the surface displayed slow electrochemical kinetics toward negatively charged species, such as [Fe­(CN)_6_]^3–^ (Figure 2d). Results presented in Figure 2e support these findings, showing enhanced electrochemical kinetics of SD-TNT in the presence of 5 mmol L^–1^ [Fe­(CN)_6_]^3–^ after applying −1.5 V for 900 s in a 1 mol L^–1^ NaClO_4_ solution, a medium in which superficial anionic adsorption does not occur, due to the absence of phosphate species in solution.

The electrochemical behavior of [Fe­(CN)_6_]^3–^ at the SD-TNT surface was investigated by varying the pH from 2.0 to 11 in BR solution (Figure S5). Initially, it was observed that the electrochemical kinetics of [Fe­(CN)_6_]^3–^ probe on the SD-TNT electrode was irreversible across all pH values. Furthermore, the peak potential (*E*_p,c_) shifted to more negative values as the pH increased (from −0.20 V at pH 2.0 to −0.49 V at pH 11) (Table S2). In a strongly acidic medium (pH = 2.0), the SD-TNT surface becomes protonated, neutralizing the phosphate species adsorbed during cathodic polarization and facilitating ferricyanide reduction on the electrode surface. In contrast, at higher pH levels, deprotonated phosphate groups result in a negatively charged surface, which impairs interactions with anionic species such as ferricyanide.

To evaluate the behavior of a positively charged electrochemical probe, CVs were obtained at different concentrations of [Ru­(NH_3_)_6_]^3+^ (0.10–10 mmol L^–1^), and different ν (0.0100–1.00 V s^–1^) were used (Figure S6). In all experiments, the reduction and oxidation peaks of the probe can be observed. However, the mass transport of the probe appears to be affected by concentration variation and scan rate.

Figure 3a presents the behavior of the log–log plot of *I*_p,c_ vs ν, while Figure 3b presents *I*_p,c_ normalized by the concentration of [Ru­(NH_3_)_6_]^3+^ as a function of ν.

**Figure 3:**
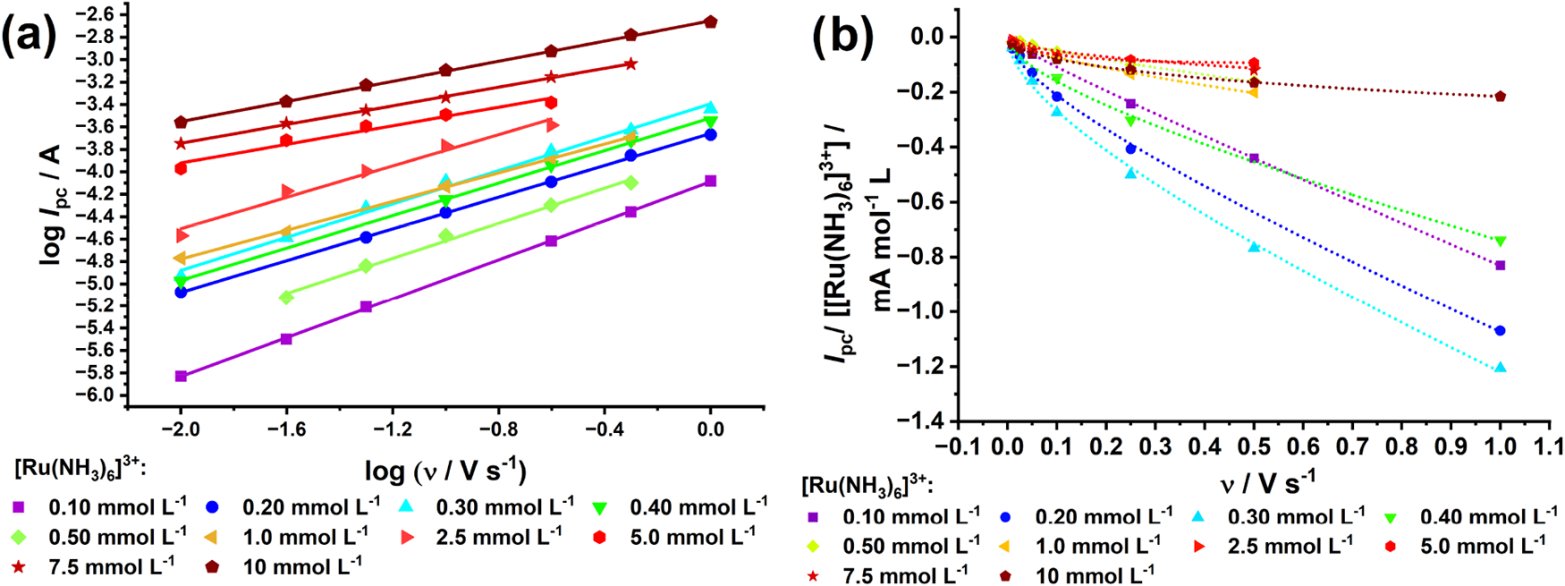
Linear fit of log *I*_p,c_ vs log ν (a) and nonlinear fit of *I*_p,c_ vs ν plot, eq 11 (b) obtained from cyclic voltammograms in 0.50 mol L^–1^ KCl and [Ru­(NH_3_)_6_]^3+^ from 0.10 to 10 mmol L^–1^ at different scan rates. Cyclic voltammograms are shown in Figure S6.

Table 2 presents the slope and *R* ^2^ values obtained from the linear fit of log­(*I*_p,c_) vs log­(ν), varying the concentration of [Ru­(NH_3_)_6_]^3+^ from 0.10 to 10 mmol L^–1^.

**Table 2:** Slope Values and *R* ^2^ Obtained from Linear Fit of log­(*I*_p,c_ vs ν), Varying the Concentration of [Ru­(NH_3_)_6_]^3+^ from 0.10 to 10 mmol L^–1^

⌈Ru(NH_3_)_6_⌉^3+^/mmol L^–1^	slope	*R* ^2^
0.10	0.87	0.999
0.20	0.71	0.999
0.30	0.74	0.993
0.40	0.72	0.998
0.50	0.78	0.991
1.0	0.64	0.999
2.5	0.70	0.972
5.0	0.41	0.954
7.5	0.42	0.999
10	0.45	0.999

A slope of 0.5 indicates that the process is diffusion-controlled, while a slope close to 1 indicates a process associated with species adsorbed on the electrode.^51^ Consequently, a value between 0.5 and 1 indicates both species diffusing from solution and adsorbed (mixed process).^52^

To study a mixed process, it is important to consider the adsorption, eq 10, along with the diffusional components, eq 9. Considering both processes lead to eq 11.^52–54^

9 $$I_{p}=av^{0.5}$$

10 $$I_{p}=\mathrm{bv}$$

11 $$I_{p}=cv^{0.5}+\mathrm{dv}$$

where ν is the scan rate and “*a*,” “*b*,” “*c*,” and “*d*” are parameters that depend on the properties of the molecules involved in the reaction, experimental conditions, and electrochemical mechanism.

Based on the data presented in Figure 3a and Table 2, the slope of the log *I*_p,c_ versus log ν relationship decreases from 0.87 to 0.41 as the concentration of [Ru­(NH_3_)_6_]^3+^ increases from 0.10 to 2.5 mmol L^–1^, remaining nearly constant (0.41–0.45) at higher concentrations. Slopes significantly higher than 0.5 at low concentrations indicate a dominant contribution from adsorbed species. As the concentration increases, the system progressively transitions toward diffusion-controlled behavior, as also supported by the nonlinear fitting of *I*_p,c_ versus ν (eq 11), where the ν^1/2^ term becomes more significant above 0.50 mmol L^–1^.

This behavior can be understood by considering the interaction between the probe and electrode surface. At low concentrations, the adsorption of the probe onto available active sites of the SD-TNT surface contributes significantly to the current response. As the concentration increases, these sites approach saturation, and the additional current arises predominantly from diffusion of [Ru­(NH_3_)_6_]^3+^ from the bulk solution to the electrode interface.^34,55^

Despite this transition toward diffusion-controlled transport, the slopes obtained at concentrations above 2.5 mmol L^–1^ remain below the theoretical value of 0.5 predicted by the Randles–Ševčík equation. This deviation arises mainly from nonideal effects associated with the increase in current. In particular, higher probe concentrations lead to larger faradaic currents, which enhance the ohmic drop (*E*′ = *E* – *R*_u_ *I*) across the uncompensated resistance *R*_u_. As a result, the effective potential at the electrode/solution interface is different than the applied potential, distorting the voltammetric response and leading to lower values of *I*_p,c_.^34,56^ Such distortion can be observed in Figure S6.

This effect is further amplified at higher scan rates, where currents are also larger. Voltammograms (Figure S6) show increasing deviation from ideal behavior with increasing ν, which is supported by Tafel analysis (Figure S7). Although the uncompensated resistance of the SD-TNT electrode is lower than that of TNT (Figure S2), its contribution remains significant at high ν and high concentrations. Consequently, the combined effect of scan rate and concentration results in sub-Randles–Ševčík behavior, even though the dominant transport regime is diffusion-controlled. In parallel, quasi-reversibility of the electrochemical reaction in high scan rates can also contribute to the decrease of the log­(*I*_p_) vs log­(ν) slope.^57^

The adsorption of [Ru­(NH_3_)_6_]^3+^ at SD-TNT endorses the idea that the SD-TNT surface is negatively charged, as inferred from the results regarding the electrochemical reaction of ferricyanide. Therefore, the detection of a positively charged analyte may be favored because of the enhanced interaction with the electrode surface. Consequently, SD-TNT was evaluated as a sensor for methylene blue, which presents a positive charge in acidic media.

### 3.3 Electrochemical Behavior of Methylene Blue Using SD-TNT Electrode

According to Scheme 1, the redox process of MB involves both protons and electrons, making it inherently pH-dependent.^58,59^ CVs recorded in MB solutions at different pH values (Figure 4a) with GCE at 0.0500 V s^–1^ indicate that the redox processes of MB become more favorable as the pH decreases (ranging from 0.095 V in pH 2.0 to −0.18 V in pH 7.4 vs Ag/AgCl, KCl­(sat.)). Nevertheless, CVs obtained using SD-TNT (Figure 4b) exhibited a more pronounced shift in *E*_p,c_ as the pH increases (from 0.036 V at pH 2.0 to −0.40 V at pH 7.4 vs Ag/AgCl, KCl­(sat.)).

**Figure 4:**
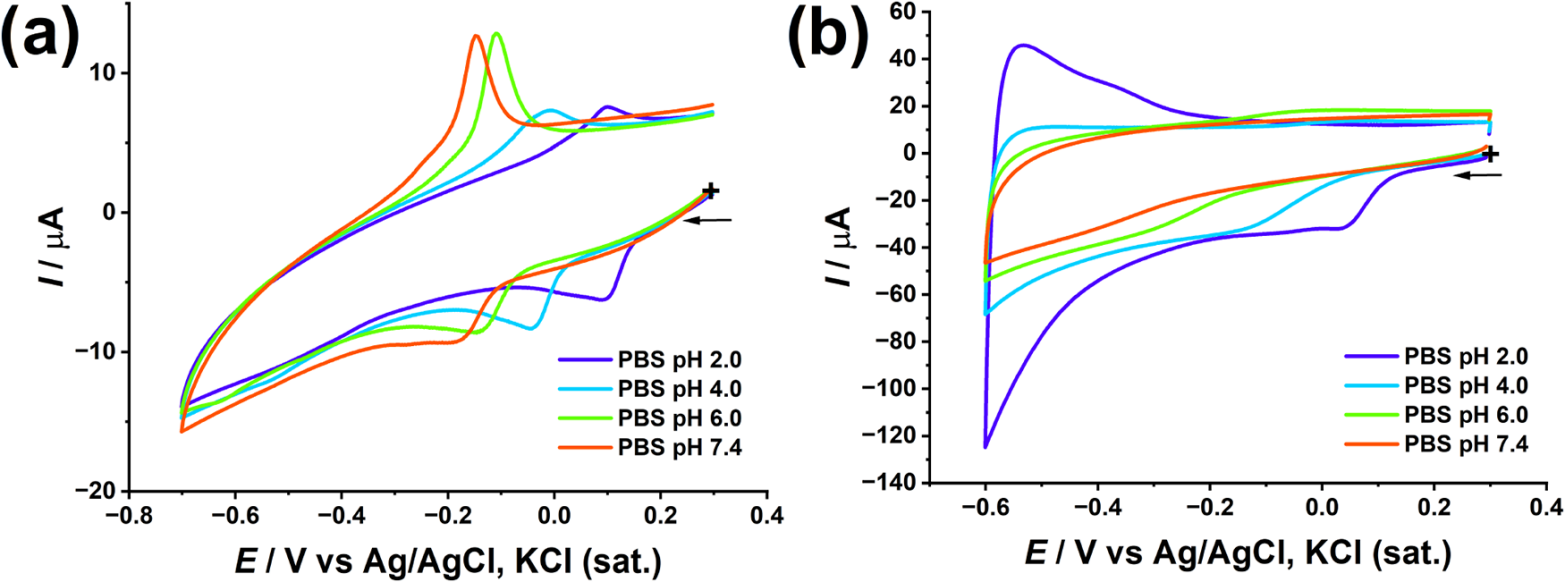
CVs in 0.25 mol L^–1^ Na_2_SO_4_ + PBS 0.1 mol L^–1^ solutions containing 0.1 mmol L^–1^ MB, using glassy carbon electrode (GCE) (a) and SD-TNT (b) at 0.050 V s^–1^ at different pH values.

**Scheme 1:**
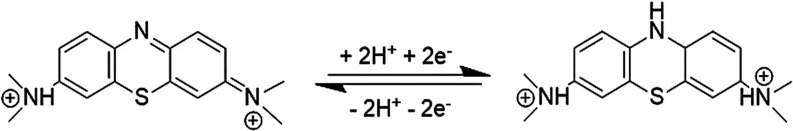
Representation of the Redox Reaction of MB^2+^ in pH 2.0

As previously confirmed, the electrochemical response of positively charged species is generally more favorable, likely because of the superficial adsorption of phosphate groups. At pH 2.0, dihydrogen phosphate and phosphoric acid exist in nearly equal proportions (p*K*_a1_ = 2.15). Consequently, some phosphate groups adsorbed to the electrode remain unprotonated. In contrast, at the same pH, MB is protonated (p*K*_a_ = 3.62, MB^2+^),^59,60^ which explains the sharper reduction peak compared with the CVs recorded at pH 4.0 and 6.0 (Figure 4b).

Figure 5a,b shows the CVs recorded in PBS pH 2.0 solutions containing 0.1 mmol L^–1^ MB^2+^ over a ν range of 0.010–1.00 V s^–1^. At lower ν (0.010 V s^–1^), a redox peak pair is observed (*E*_p,c_ = 0.075 V and *E*_p,a_ = 0.19 V), which shifts to *E*_p,c_ = 0.055 V and *E*_p,a_ = 0.25 at 0.050 V s^–1^ (vs Ag/AgCl, KCl (sat)), Figure 5a. However, as the scan rate increases, the voltammetric profiles become progressively distorted, significantly impairing quantitative assessment of peak parameters.

**Figure 5:**
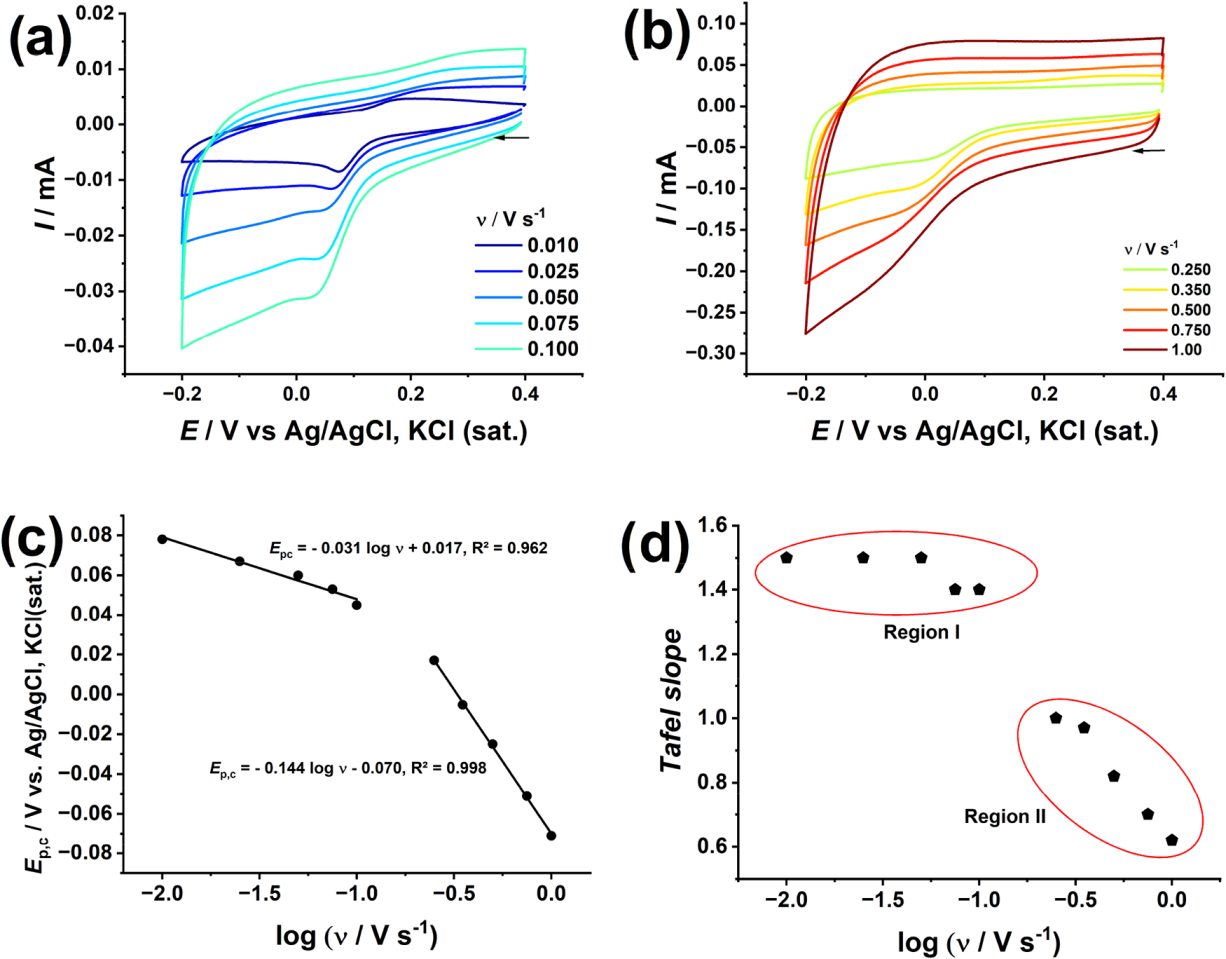
CVs recorded in 0.25 mol L^–1^ Na_2_SO_4_ + PBS 0.1 mol L^–1^ (pH 2.0) containing 0.1 mmol L^–1^ MB^2+^ (0.010–1.00 V s^–1^) using SD-TNT (a, b). Plot of *E*_p,c_ vs log ν (c). Tafel slope obtained from CVs (d).

*E*_p,c_ was plotted as a function of the logarithm of ν. Figure 5c reveals two distinct regions. In the first region (ν ≤ 0.100 V s^–1^), a linear relationship is observed with a slope of 31 mV dec^–1^.^34,55,61^ Although Scheme 1 represents an overall 2-electron/2-proton process, concerted multielectron transfers are highly improbable for organic molecules. A stepwise mechanism should therefore be assumed.^34,55,62^ To probe the rate-determining step (RDS) of the MB reduction, Tafel slopes were extracted according to eq 12. For an irreversible, stepwise, multielectronic reaction, Tafel slopes can be interpreted as the sum of *n*′, which is the number of electrons transferred prior to the RDS, and α_RDS_, which is the charge-transfer coefficient of the rate-determining step, ranging from 0 to 1 ^34,63^

12 $$n^{\prime }+\alpha_{\mathrm{RDS}}=-\frac{\mathrm{RT}}{F}\frac{\partial \ln\left(I\right)}{\partial E}$$

In eq 12, *R* is the universal gas constant (in J K^–1^ mol^–1^), *T* is the temperature in *K*, and *F* is the Faraday constant (in C mol^–1^). The analysis of voltammetric data according to eq 12 was restricted to the “foot” of the voltammetric peak, with current values up to 20% of the cathodic peak current to avoid the effect of reactant depletion at the electrode surface on the measurement.^64^

Within 0.010–0.100 V s^–1^, the Tafel slopes remain nearly constant at 1.5, pointing to the second electron transfer as the RDS. Relative to a mechanism in which the first electron transfer is rate-determining (*n*′ = 0, α_RDS_ ≈ 0.5), this regime produces higher and sharper cathodic peaks, as both peak current and peak sharpness increase with *n*′ + α_RDS_.^34,55^

For scan rates above 0.100 V s^–1^, the slope of *E*_p_ vs log ν increases to 144 mV dec^–1^, as shown in Figure 5c. However, as evidenced by Figure 5b, at these scan rates, the voltammetric peak becomes severely distorted by capacitive current and ohmic drop contributions, precluding reliable extraction of kinetic parameters. The transition between regions therefore marks the practical upper limit of scan rates within which the Tafel analysis remains valid, rather than a mechanistic change in the rate-determining step.

The scan rate range of 0.010–0.100 V s^–1^, within which the electrode kinetics is well-defined, is compatible with differential pulse voltammetry (DPV),^65^ which additionally mitigates the capacitive current contributions to the voltammetric response. Thus, DPV was chosen for the construction of the analytical curve for MB with SD-TNT.

### 3.4 Determination of Methylene Blue Using SD-TNT Electrode

CVs at 0.100 V s^–1^ in 0.25 mol L^–1^ Na_2_SO_4_ and 0.1 mol L^–1^ PBS (pH 2.0) containing 0.1 mmol L^–1^ MB solutions were performed with bare Ti, amorphous TNT, anatase TNT, and SD-TNT electrodes (Figure 6a). It can be observed that the CVs obtained with bare Ti and amorphous TNT electrodes did not show any MB^2+^ reduction or oxidation peaks. In contrast, the anatase TNT and SD-TNT electrodes exhibited reduction peaks at −0.093 and 0.051 V, respectively, with a potential shift of 144 mV. The SD-TNT electrode clearly shows an increase in the integrated area of the CV relative to the other electrodes due to enhanced capacitance as previously discussed. Additionally, the shift of the MB reduction peak to a less negative potential (0.051 V) further supports the existence of a significant interaction between MB^2+^ and the surface of the SD-TNT electrode.

**Figure 6:**
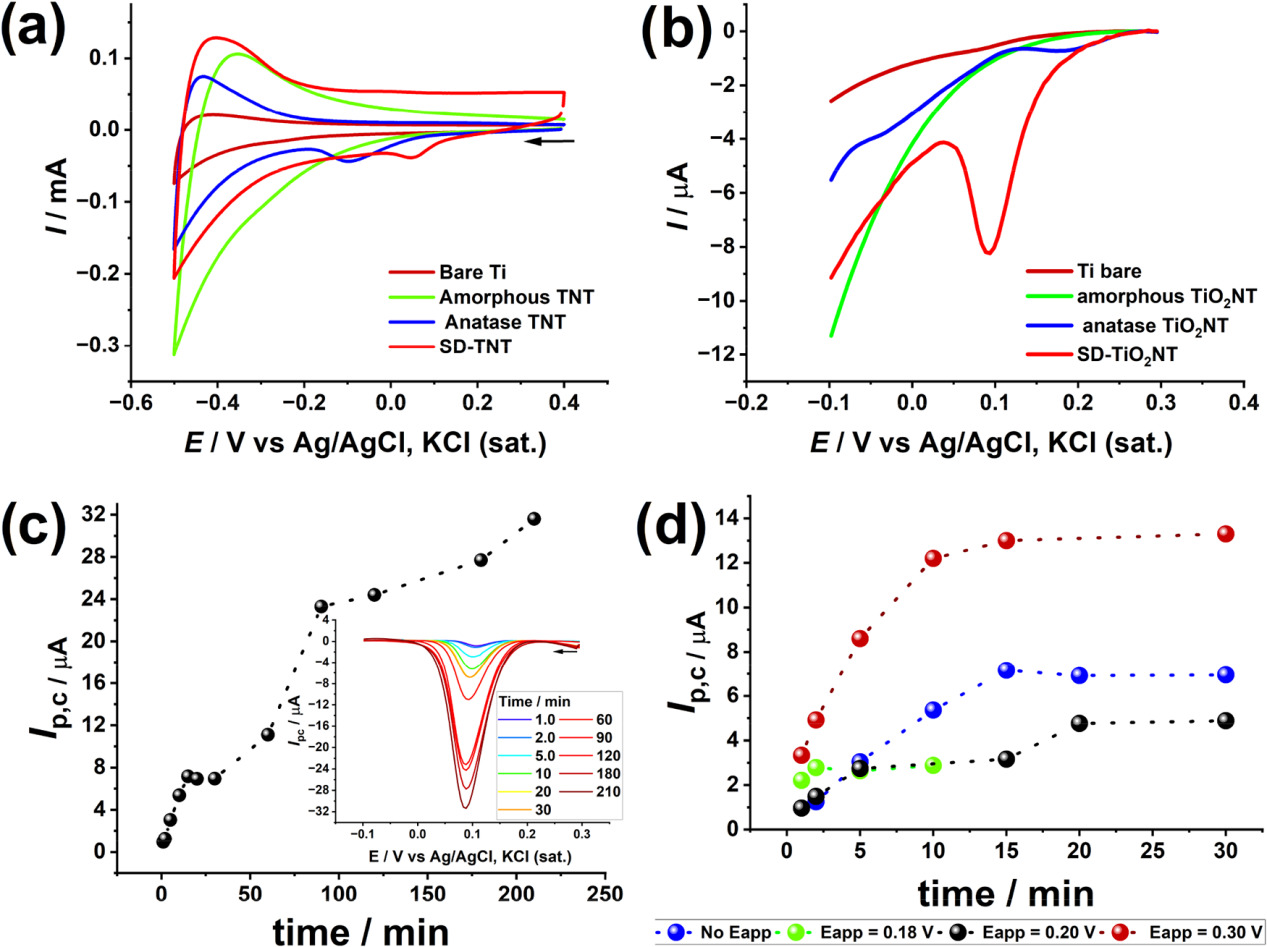
CVs obtained in 0.5 mol L^–1^ Na_2_SO_4_ + 0.1 mol L^–1^ PBS (pH 2.0) solutions containing 0.1 mmol L^–1^ MB^2+^ at 0.100 V s^–1^ using SD-TNT electrode (a). DPVs obtained with a modulation amplitude: 0.025 V, step potential of −0.005 V, and scan rate: 0.010 V s^–1^ (b). *I*_p,c_ vs preconditioning times plot (no applied potential) obtained with an SD-TNT electrode. Inset: DPV obtained in 0.1 mmol L^–1^ MB^2+^ using SD-TNT (c). *I*_p,c_ values obtained at SD-TNT electrode by DPV at different deposition times in 0.1 mmol L^–1^ MB^2+^ without applied potential and at 0.18, 0.20, and 0.30 V applied potential (d).

As a proof of concept, the SD-TNT electrode was evaluated for the electrochemical detection of methylene blue (MB) in aqueous medium. Differential pulse voltammograms (DPVs) were carried out in a 0.1 mmol L^–1^ MB solution at a scan rate of 0.010 V s^–1^. Peak currents in the microampere range were obtained, demonstrating the superior performance of the SD-TNT electrode in comparison to other titanium-based electrodes, Figure 6b.

Differential pulse voltammetry was performed at the SD-TNT electrode in deaerated 0.1 mmol L^–1^ MB^2+^ under the same conditions as described above (Figure 6c). In the initial stages (1–5 min), a rapid adsorption of MB^2+^ onto the SD-TNT surface is observed (Figure S8). This behavior is attributed to the high availability of active sites on the electrode surface. The first plateau region of the *I*_p,c_ can be observed in Figure 6c (10–30 min), which possibly corresponds to the formation of the initial monolayer of MB^2+^ on the SD-TNT surface.^59,66,67^ After 30 min, a gradual increase in *I*_p,c_ is observed and increases in the direction of the second plateau although at a slower rate (Figures 6c and S8). This phenomenon is characteristic of cooperative adsorption,^68^ in which the initial MB^2+^ layer formed on the SD-TNT surface facilitates the adsorption of additional MB^2+^ species from the bulk solution.^69–71^

SD-TNT was preconditioned at different potentials (0.18–0.30 V), before each electrochemical measurement into MB^2+^, to stabilize the response of MB^2+^ at the SD-TNT surface (Figure 6d). The *I*_p,c_ values remained nearly constant over time, stabilizing within the first 2 min at *E*_app_ = 0.18 V (Figure S10). However, these values decreased by a factor of 2.5 than those observed in differential pulse voltammetry studies conducted without an applied potential. This suggests that at *E*_app_ = 0.18 V, there was a depletion of MB^2+^ species at the electrode–solution interface due to the application of a potential sufficiently close to the reduction potential of MB^2+^.

In contrast, at *E*_app_ = 0.30 V, the applied potential is not near the MB^2+^ potential reduction potential, but the substantial increase in the current levels can be attributed to the phosphate additional adsorption, which promotes the MB^2+^ adsorption involving the substitution of the solvated water molecules by target adsorption at the electrode surface.^72–75^ *I*_p,c_ stabilizes after 10 min (Figure S9c), showing a 2.5-fold increase compared to the experiments performed without an applied potential.

Similar experiments, under the same conditions, were performed substituting phosphate with perchlorate ions during all steps of the SD-TNT preparation. The cathodic polarization step was conducted by applying −1.5 V for 15 min in 1 mol L^–1^ NaClO_4_ solution. Although a reduction peak can still be observed at 0.1 V, the signal is noticeably weaker than that obtained for the electrode modified in the presence of phosphate (Figure S10). This behavior suggests that, unlike phosphate species, perchlorate ions do not promote significant superficial modification, and the MB^2+^ preconcentration step was not observed.

Analytical curves (Figure 7a) were obtained using SD-TNT and differential pulse voltammetry. The concentration of MB in the solutions ranged from 1.0 × 10^–5^ to 9.0 × 10^–5^ mol L^–1^. The *I*_p,c_ exhibits a linear correlation with MB concentration in the range of 1.5–6.0 × 10^–5^ mol L^–1^, following the equation *I*_p,c_ = −0.118 μA L mol^–1^ × [MB] + 1.52 μA (*R* ^2^ = 0.9991). However, at concentrations above 6.0 × 10^–5^ mol L^–1^, a deviation from linearity was observed, as the current response becomes more negative than expected. This behavior confirms a cooperative adsorption phenomenon, likely driven by the interaction between MB^2+^ adsorbed on the SD-TNT surface. This interaction occurs through π–π stacking between the aromatic rings of MB molecules, which is higher as their concentration in the bulk solution increases.^76,77^

**Figure 7:**
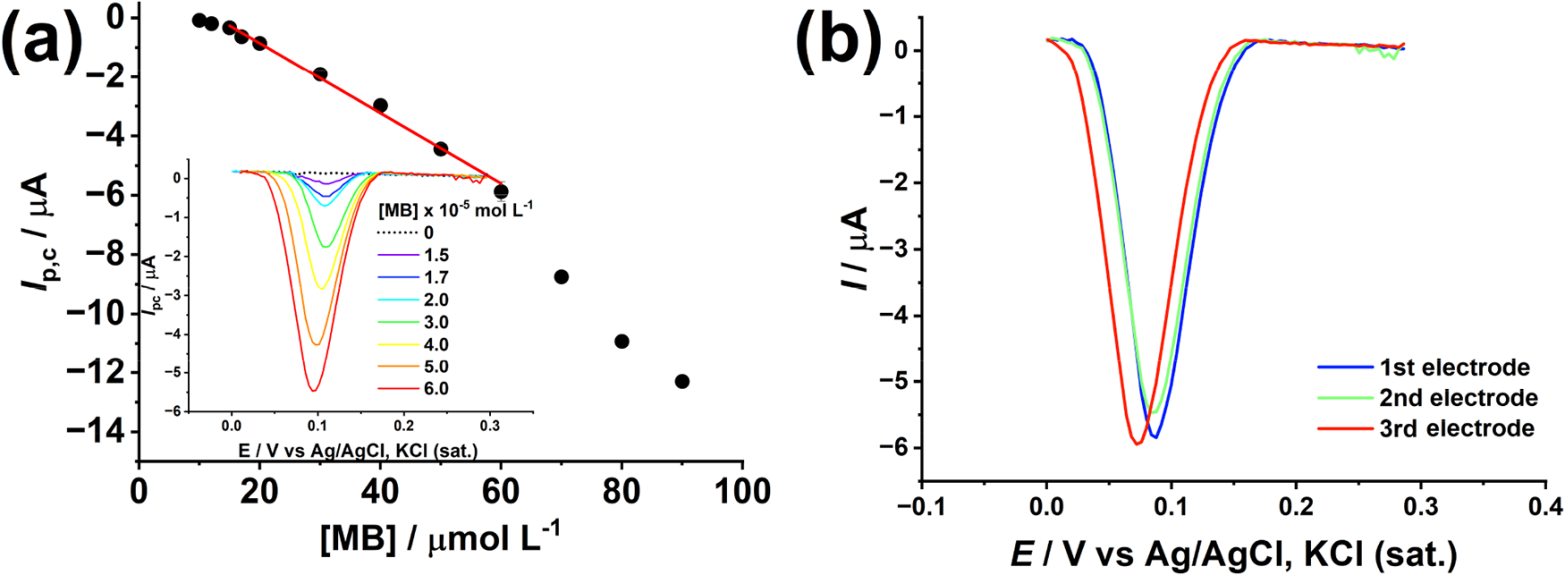
Analytical curve obtained at SD-TNT in 0.5 mol L^–1^ Na_2_SO_4_ + 0.1 mol L^–1^ PBS, pH 2.0 containing MB^2+^ in the concentration range of 1.5–6.0 × 10^–5^ mol L^–1^. Measurements were performed in duplicate using a deaerated solution. Inset: DPVs obtained using the SD-TNT electrode after applying a potential of 0.30 V for 10 min (a). DPVs obtained at 0.010 V s^–1^ using three different SD-TNT electrodes in 0.5 mol L^–1^ Na_2_SO_4_ + 0.1 mol L^–1^ PBS (pH = 2.0) containing 6.0 × 10^–5^ mol L^–1^ MB^2+^, after 0.30 V applied potential for 10 min (b). Equation of the analytical curve: *I*_p,c_ = −0.118 μA L mol^–1^ × [MB] + 1.52 μA *R* ^2^ = 0.9991.

The calculated LOD and LOQ for MB^2+^ were 0.202 and 0.674 μmol L^–1^, respectively. The reproducibility of SD-TNT electrodes was assessed by fabricating three independent sensors under the same conditions and evaluating their responses toward 6.0 × 10^–5^ mol L^–1^ MB (Figure 7b). The average *E*_p,c_ and *I*_p,c_ values obtained from DPV measurement were 81.9 ± 7.35 mV and −5.89 ± 0.20 μA, respectively, corresponding to relative standard deviation (RSDs) of 7.35% for *E*_p,c_ and 3.43% for *I*_p,c_, thereby demonstrating good fabrication reproducibility.

As summarized in Figure 7 and Table 3, the SD-TNT electrode exhibited a lower LOD compared to other Ti-based and composite electrodes reported in the literature.

**Table 3:** Comparison of SD-TNT with Electrochemical Sensors for the Determination of MB Reported in the Literature

analytical technique	type of electrode	LOD μmol L^–1^	references
CV	SD-TNT	0.900	^21^
CV	NiO-NCQD/g-C_3_N_4_/rGO/CPME	0.410	^78^
SWV	thiol-functionalized clay	0.400	^79^
EIS	SD-TNT	0.313	^42^
**DPV**	**SD-TNT**	**0.202**	**this work**

Beyond the analytical figures of merit summarized in Table 3, the SD-TNT electrode offers distinctive morphological and electrochemical advantages over the other platforms. The vertically aligned nanotube architecture provides a high surface-to-volume ratio that favors MB^2+^ accumulation at the interface without requiring external modifiers or binders. Unlike composite electrodes, such as NiO-NCQD/g-C_3_N_4_/rGO/CPME and thiol-functionalized clay, SD-TNT is synthesized using low-cost, less toxic solvents through a relatively straightforward methodology. Electrochemically, the combination of Ti­(III) states and phosphate-induced surface charge distinguishes SD-TNT from conventional TiO_2_-based platforms, enabling both enhanced charge transfer and electrostatic preconcentration of MB^2+^.

## 4 Conclusion

Self-doped TiO_2_ nanotube (SD-TNT) electrodes, prepared by cathodic polarization in phosphate buffer, exhibited pronounced structural and electrochemical modifications associated with the formation of Ti­(III) states and the adsorption of phosphate species onto the nanotube surface. These superficial changes enhanced electrical conductivity and surface hydrophilicity, creating a favorable environment for MB^2+^ accumulation under acidic conditions. The electrochemical behavior of MB^2+^ on SD-TNT electrodes was pH-dependent. The redox process was more favorable under an acidic medium due to the preponderant MB^2+^ species in solution, which is preconcentrated at the negatively charged electrode surface. Scan rate studies revealed distinct kinetic regimes. Within 0.010–0.100 V s^–1^, Tafel analysis identified the second electron transfer as the rate-determining step. At higher scan rates, capacitive current and ohmic drop contributions distorted the voltammetric response, precluding reliable kinetic analysis. As proof of concept, the modified electrode was further employed for the electrochemical detection of MB in acidic medium, showing a promising limit of detection (0.202 μmol L^–1^) and reproducibility (RSDs = 7.35% for *E*_p,c_ and 3.43% for *I*_p,c_).

## Supplementary Material



## Notes

Cyclic voltammograms recorded for TNT and SD-TNT electrodes in 0.5 mol L^–1^ H_2_SO_4_ at varying scan rates; simulated data of uncompensated resistance; cyclic voltammograms of TNT and bare Ti at varying pH; semidifferential cyclic voltammograms obtained for [Ru­(NH_3_)_6_]^3+^ and [Fe­(CN)_6_]^3–^ probes; cyclic voltammograms of SD-TNT in [Fe­(CN)_6_]^3–^ solution at pH 2–11 and corresponding *E*_p,c_ values; cyclic voltammograms of SD-TNT in [Ru­(NH_3_)_6_]^3+^ solutions at different concentrations and scan rates; Tafel analysis derived from CVs using [Ru­(NH_3_)_6_]^3+^; first derivative of the *I*_p,c_ vs contact time curve obtained from DPV in 0.1 mmol L^–1^ MB using SD-TNT; DPV responses obtained at different deposition potentials; DPV signals recorded after 10 min of preconcentration with and without PBS (PDF)

## Abbreviations

MB: methylene blue
TNT: nanotube of TiO_2_
SD-TNT: nanotube of TiO_2_ self-doped
CVs: cyclic voltammograms
DPVs: differential pulse voltammograms
