# Supplementary material for: Interfacial Chemistry of Phosphate-Functionalized Self-Doped TiO2 Nanotube for Electrochemical Detection of Methylene Blue
Source: ACS Omega. 2026 Jun 2;11(24):35776–88. doi: 10.1021/acsomega.6c02245 (PMC13294902; doi:10.1021/acsomega.6c02245)
Supplement: Supplementary file 1 [file ao6c02245_si_001.pdf]

Supporting Information

Interfacial chemistry of phosphate-functionalized self-doped TiO_2 nanotube for electrochemical detection of methylene blue

Victor Lorejan Pinto[1], Rafael A. L. Chioquetti[2], Silvia H. P. Serrano[3]*.

Institute of Chemistry, Department of Fundamental Chemistry, University of Sao Paulo, Prof. Lineu Prestes Avenue, 784, Butanta, 05508-900, São Paulo, Brazil.

Email address: victor.lorejan@usp.br[1], rafaelchioquetti@usp.br[2], shps@iq.usp.br[3].

** Corresponding author.*

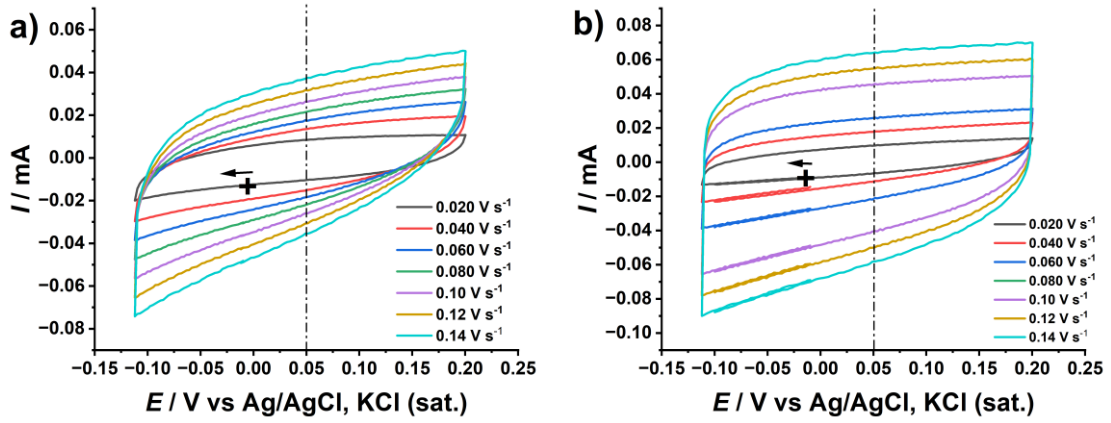

Figure S1. Cyclic voltammograms of TNT (a) and SD-TNT (b) in 0.5 mol L^{-1} H_2SO_4 with increasing scan rate.

Simulated data by the uncompensated resistance (R_u) were generated by varying R_u from 0 to 5,000 Ω (Figure S2). For R_u = 0 Ω, the resulting cyclic voltammograms (CVs) exhibit a 90° shape, whereas increasing R_u produces progressively more flattened CVs (angle < 90°). A visual analysis of CVs for TNT and SD-TNT (Fig. S1), reveals that cathodic polarization decreases the resistance of the SD-TNT electrode. This is evidenced by the SD-TNT CV profiles, which approach the 90° shape typical of less resistive electrodes, while the TNT profiles are more flattened, consistent with the behavior of more resistive electrode.

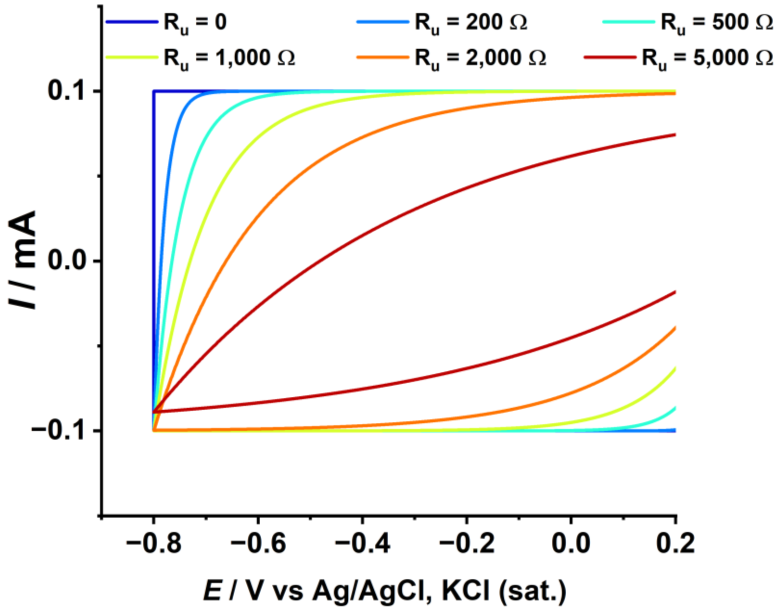

Figure S2. CVs obtained from simulated data by varying the uncompensated resistance (R_u) value.

To identify the titanium reduction and oxidation peaks present at the electrode surface, measurements using bare Ti were performed under the same experimental conditions applied to the TNT electrode. Prior to each voltammetric cycle, a potential of 3.0 V was applied to the electrode for 300 s to oxidize Ti(III) species that had been reduced during the electrochemical process (Figure S3).

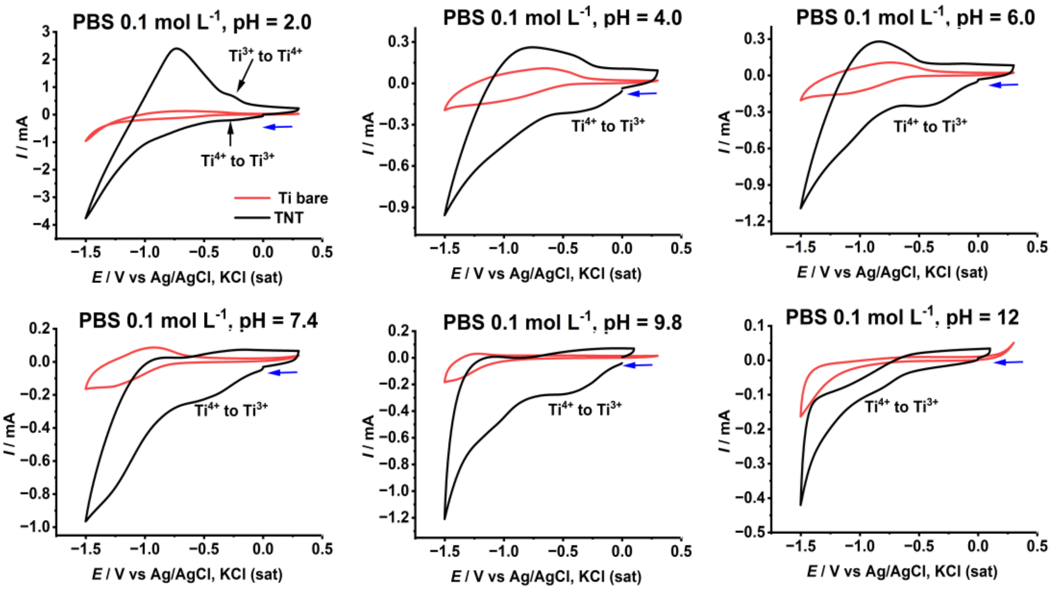

Figure S3. CVs obtained using TNT **(black line)** and and Ti bare **(red line)** electrodes after preconditioning at 3.0 V potential during 300 s in 0.1 mol L^{-1} PBS buffer (pH 2.0, 4.0, 6.0, 7.4, 9.8 and 12) at 0.100 V s^{-1}.

The semi-derivative analysis (Figure S4) was performed on the CVs of $[Ru(NH_3)_6]^{3+}$ (Figure 2c) and $[Fe(CN)_6]^{3-}$ (Figure 2d). This analysis allows the transformation of the characteristic voltametric shape into a simple mathematical curve with peaks that are easier to visualize and to fit mathematically.[1–3]

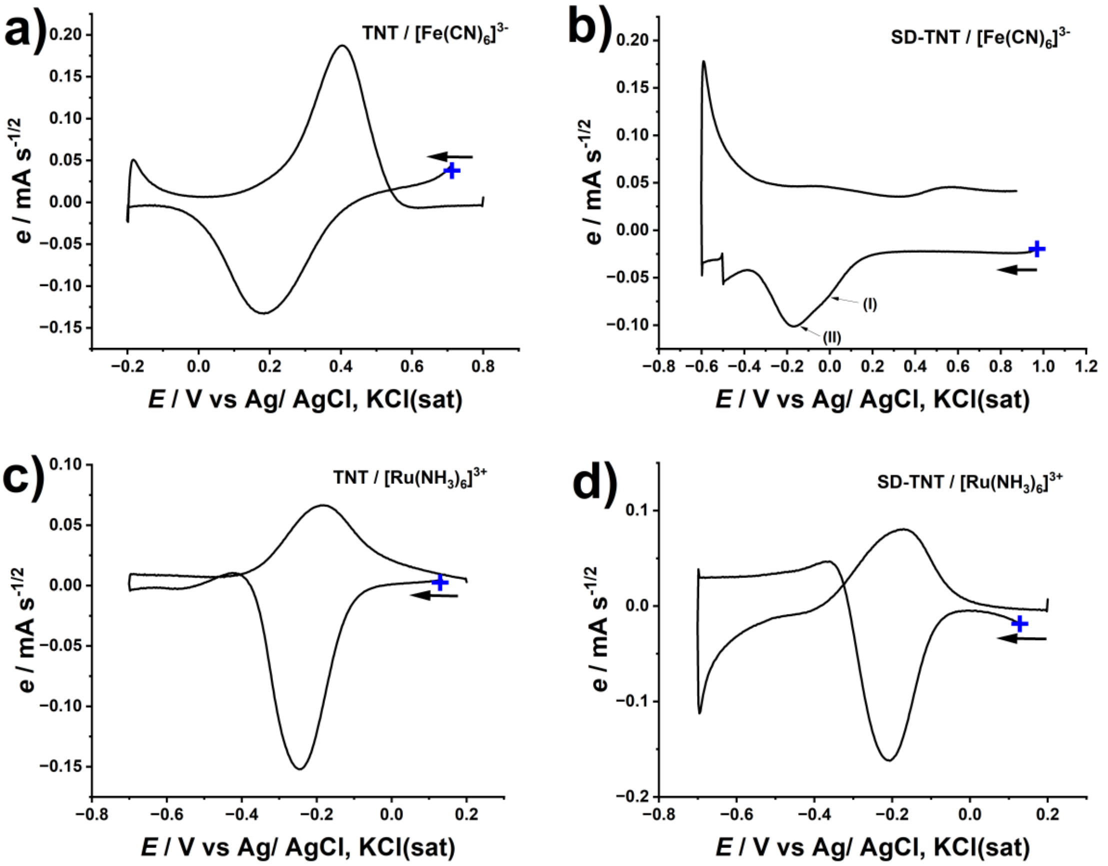

Figure S4. Semi-differentiated CVs obtained in 0.5 mol L^{-1} KCl containing 5 mmol L^{-1} $[Fe(CN)_6]^{3-}$ using TNT (a) and SD-TNT (b) as working electrode, and containing 5 mmol L^{-1} $[Ru(NH_3)_6]^{3+}$ using TNT (c) and SD-TNT (d) as working electrode at 0.1 V s^{-1}. The original cyclic voltammograms are presented in Figures 2c and 2d of the main manuscript.

For the CVs obtained in SD-TNT electrode in a solution of $[Fe(CN)_6]^{3-}$, the semi-derivative analysis reveals two overlapped peaks (I and II, Figure S4b). This split-peak behavior in CV for single-electron transfer reaction may be related to the electrochemical heterogeneity of the electrode.[4] Reports in the literature indicate that the self-doping process results in the reduction of approximately 7% of Ti(IV) to Ti(III).[5] Consequently, the formation of Ti^{3+} active sites on the TiO_2 substrate generates small "electroactive islands" that are kinetically distinct from the substrate.[4] In this context, since SD-TNT is kinetically heterogeneous, ferricyanide would display a more or less irreversible behavior depending on whether probe depletion occurs at Ti (III) active sites or on the non-self-doped substrate. Thus, these events would take place at different potentials, as evinced by the appearance of two processes (Figure S4b). The effect of electrode surface heterogeneity was also observed by Chioquetti et al., (2025),[3] when working with

modified carbon paste electrode for the determination of adenosine. Furthermore, semi-derivative analysis reveals CV profiles characteristic of adsorbed electroactive species (Figure S4c and S4d).[6,7]

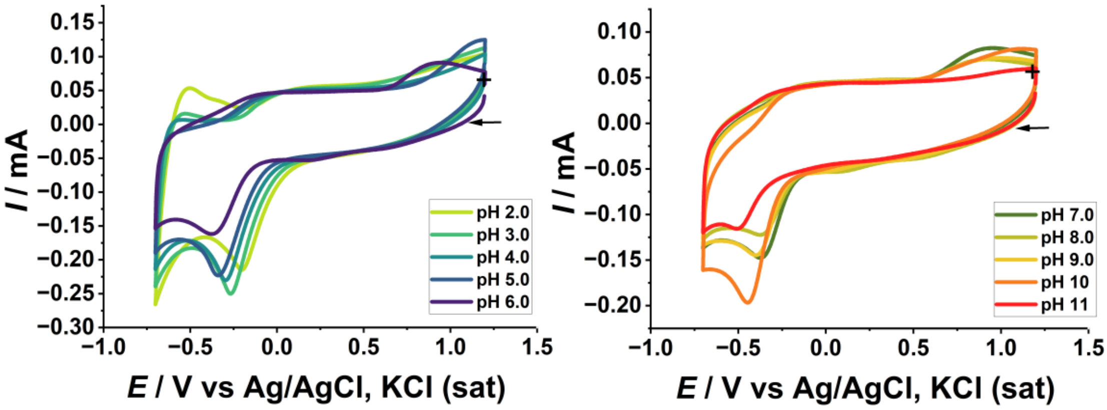

Figure S5. CVs obtained at SD-TNT in 0.2 mol L^{-1} Britton-Robinson buffer solutions (pH 2.0 - 11) containing 5 mmol L^{-1} $[Fe(CN)_6]^{3-}$. Scan rate of 0.100 V s^{-1}.

Table S2. Values of $E_{c,p}$ for each CV obtained in Britton-Robinson 0.2 mol L^{-1} (pH 2.0 - 11) solutions, containing 5 mmol L^{-1} $[Fe(CN)_6]^{3-}$ at scan rate of 0.100 V s^{-1}

pH	$E_{c,p}$ / V	pH	$E_{c,p}$ / V
2.0	- 0.20	7.0	- 0.36
3.0	- 0.26	8.0	- 0.36
4.0	- 0.29	9.0	- 0.39
5.0	- 0.34	10	- 0.43
6.0	- 0.36	11	- 0.49

Figure S6 shows the CVs obtained at SD-TNT in 0.5 mol L^{-1} KCl containing (0.10 to 10 mmol L^{-1}) $[Ru(NH_3)_6]^{3+}$ concentrations at variable scan rates. To identify the reduction and oxidation peaks of the probe, cyclic voltammograms in KCl 0.5 mol L^{-1} were subtracted.

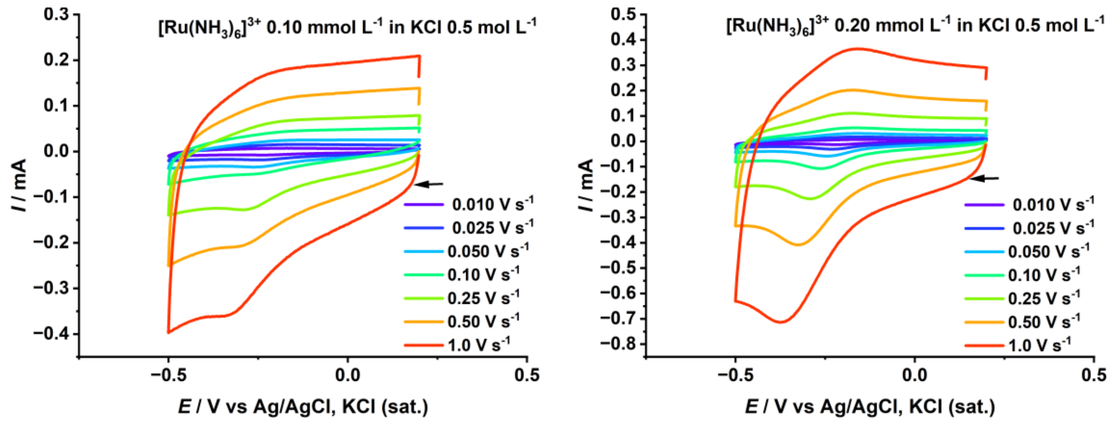

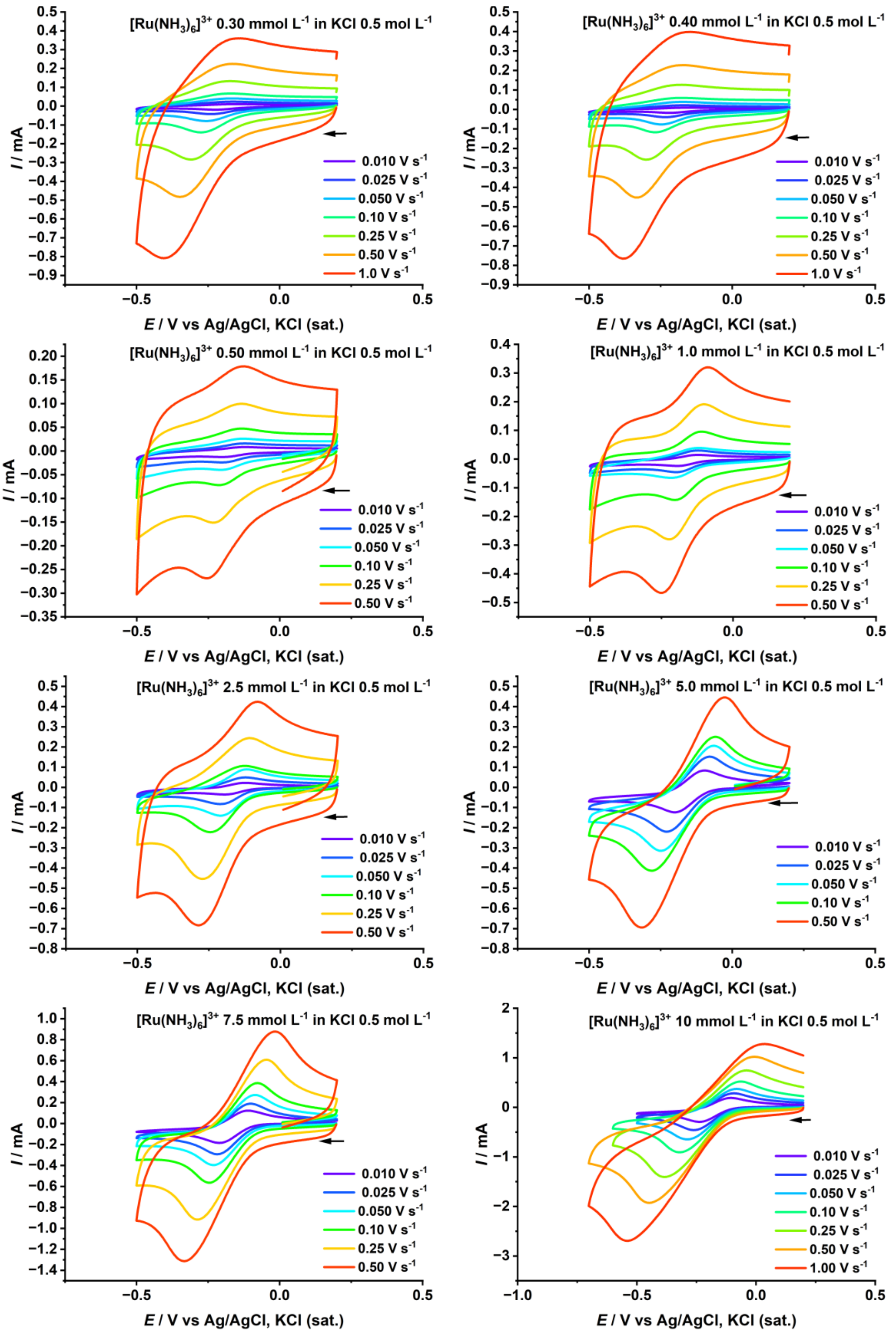

Figure S6. CVs obtained at SD-TNT in 0.5 mol L^{-1} KCl containing 0.10 to 10 mmol L^{-1} $[Ru(NH_3)_6]^{3+}$ at 0.010 to 1.0 V s^{-1}, after a previous CVs at 0.5 mol L^{-1} H_2SO_4 to oxidize $[Ru(NH_3)_6]^{3+}$adsorbed on the electrode surface.

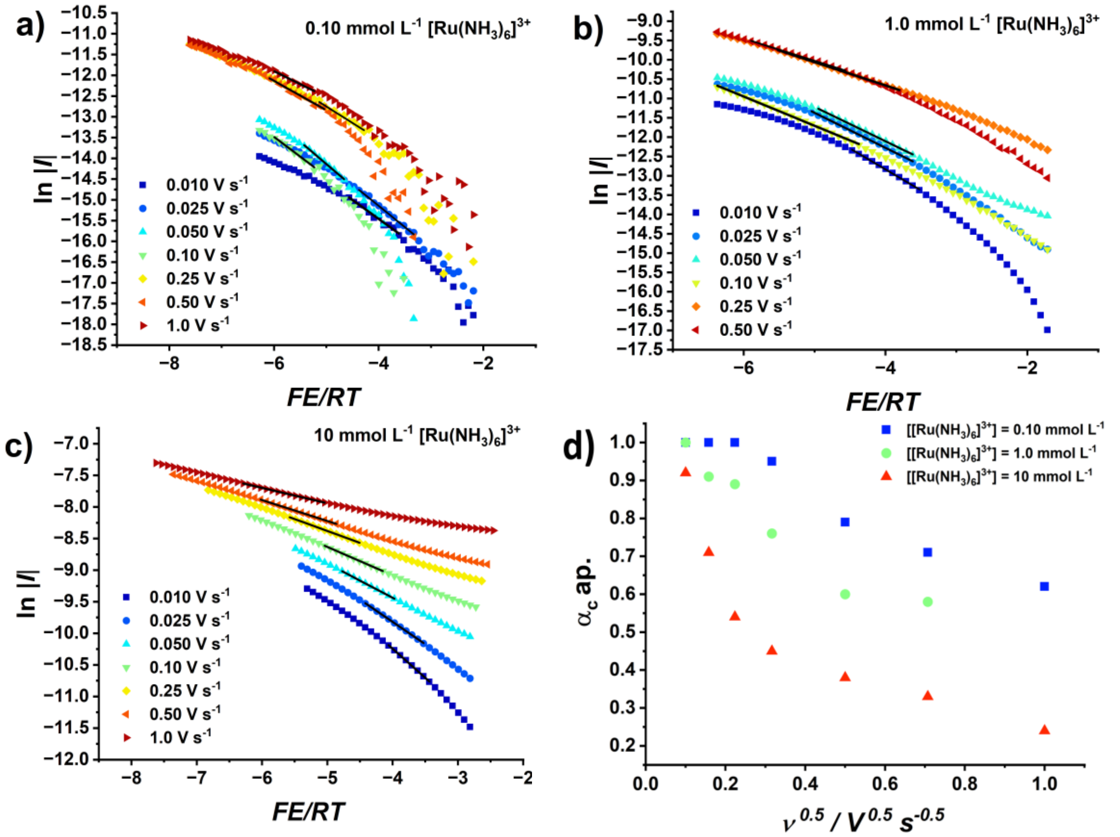

Figure S7. Tafel analysis CV obtained in KCl 0.5 mol L^{-1} and 0.10 (a), 1.0 (b), and 10 mmol L^{-1} (c) of $[Ru(NH_3)_6]^{3+}$using SD-TNT electrode. Panel (d) shows the Tafel slopes resulting from the analyses.

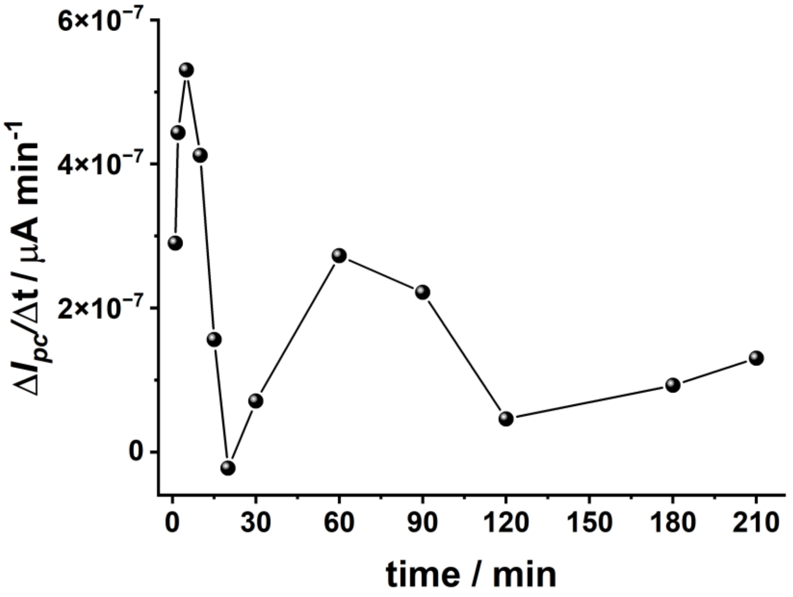

Figure S8. First derivative of the $I_{p,c}$ vs. contact time curve for 0.1 mmol L^{-1} MB solution using the SD-TNT electrode.

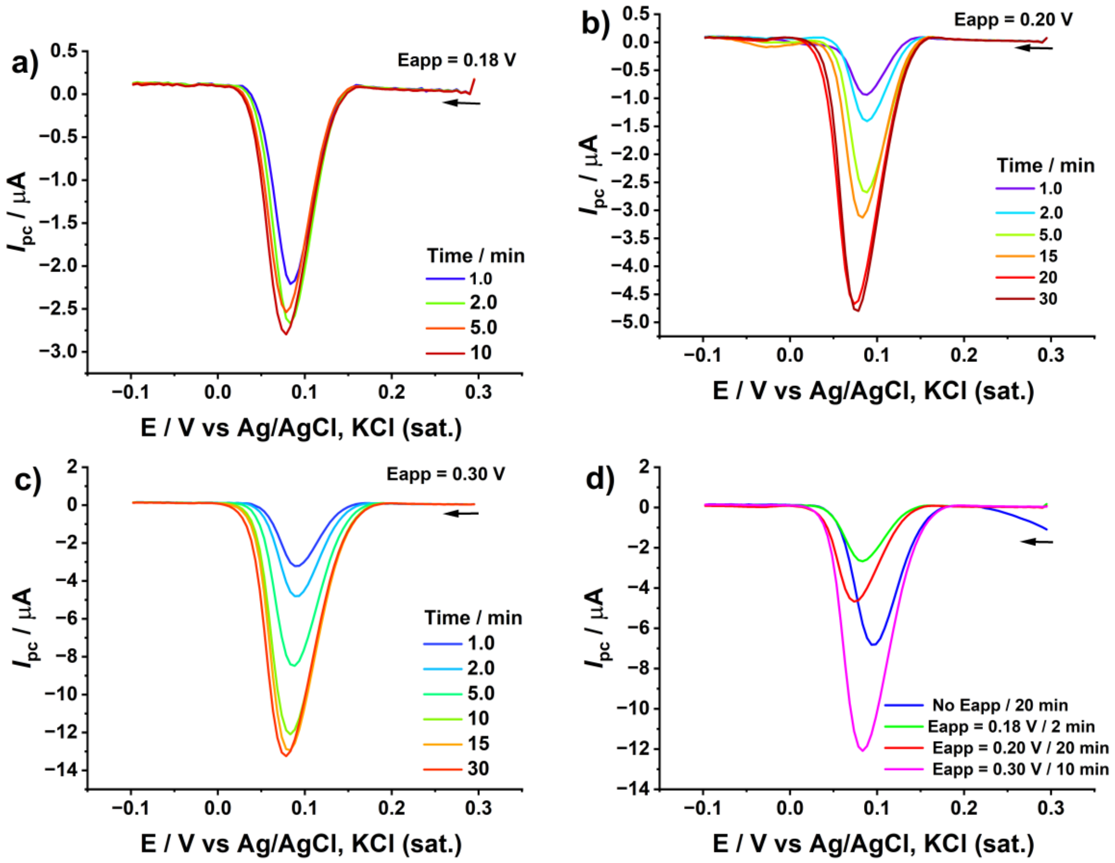

Figure S9. DPVs obtained with SD-TNT electrode in 0.5 mol L^{-1} Na_2SO_4 + 0.2 mol L^{-1} PBS (pH 2.0) containing 0.1 mmol L^{-1} MB at 0.18 V **(a)**, 0.20 V **(b),** and 0.30 V **(c)** deposition potentials. Modulation amplitude: 0.025 V; step potential: - 0.005 V; scan rate: 0.010 V s^{-1}. Optimization of the $I_{p,c}$ values were obtained at different deposition potentials **(d).**

Figure S10 compares DPVs obtained using SD-TNT in 0.1 mmol $L^{-1}$$MB^{2+}$, at two different supporting electrolytes.

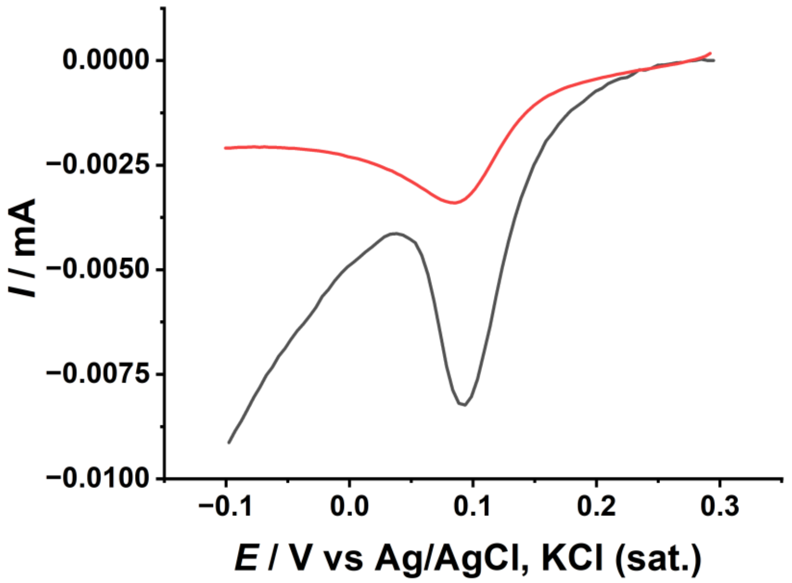

Figure S10. DPVs obtained with SD-TNT in 0.5 mol L^{-1} Na_2SO_4 + 0.2 mol L^{-1} PBS pH 2.00 containing 0.1 mmol L^{-1} MB^{2+} after 10 min of preconcentration at 0.30 V (**black line**) and in 0.5 mol L^{-1} $NaClO_4$ pH 2.0 containing 0.1 mmol L^{-1} MB^{2+} (**red line**). Prior to the DPV measurements, the SD-TNT was subjected to cathodic polarization at -1.5 V for 15 min in the respective supporting electrolytes.

References

(1) Grdeń, M. Semi-Differential Analysis of Irreversible Voltammetric Peaks. *Journal of Solid State Electrochemistry* **2017**, *21* (4), 1045–1058. https://doi.org/10.1007/s10008-016-3461-7.

(2) Mahon, P. J.; Oldham, K. B. Semioperations and Convolutions in Voltammetry. *ChemElectroChem*. Wiley-VCH Verlag March 1, 2018, pp 839–848. https://doi.org/10.1002/celc.201701167.

(3) Chioquetti, R. A. de L.; da Silva, D. P. C.; Serrano, S. H. P. Modification of Carbon Paste Electrodes for the Selective Determination of Adenosine in the Presence of Phosphate Adenylic Derivatives. *Journal of Solid State Electrochemistry* **2025**, *29* (3), 1105–1120. https://doi.org/10.1007/s10008-024-05972-w.

(4) Ward, K. R.; Lawrence, N. S.; Hartshorne, R. S.; Compton, R. G. The Theory of Cyclic Voltammetry of Electrochemically Heterogeneous Surfaces: Comparison of Different Models for Surface Geometry and Applications to Highly Ordered Pyrolytic Graphite. *Physical Chemistry Chemical Physics* **2012**, *14* (20), 7264–7275. https://doi.org/10.1039/c2cp40412e.

(5) Zhang, Z.; Hedhili, M. N.; Zhu, H.; Wang, P. Electrochemical Reduction Induced Self-Doping of Ti3+ for Efficient Water Splitting Performance on TiO2 Based

Photoelectrodes. *Physical Chemistry Chemical Physics* **2013**, *15* (37), 15637–15644. https://doi.org/10.1039/c3cp52759j.

(6) Klic’ Ka, R. *Adsorption in Semi-Differential Voltammetry*; 1998; Vol. 455.

(7) Bernard, M. O.; Bureau, C.; Soudan, J. M.; Lfcayon, G. *Diagnosis of Adsorption on Solid Electrodes Using Semi-Integral Voltammetry: A Generalization via Asymptotic Analysis*; 1997; Vol. 431.
